# Visualizing the distribution of various inorganic metals in brown rice by radiotracer experiments

**DOI:** 10.1093/mtomcs/mfag006

**Published:** 2026-03-06

**Authors:** Atsushi Hirose, Tomoko M Nakanishi, Keitaro Tanoi, Natsuko I Kobayashi

**Affiliations:** Department of Pharmacology, Hoshi University, 2-4-41 Ebara, Shinagawa-ku, Tokyo 142-8501, Japan; Graduate School of Agricultural and Life Sciences, The University of Tokyo, 1-1-1 Yayoi, Bunkyo-ku, Tokyo 113-8657, Japan; Graduate School of Agricultural and Life Sciences, The University of Tokyo, 1-1-1 Yayoi, Bunkyo-ku, Tokyo 113-8657, Japan; Graduate School of Agricultural and Life Sciences, The University of Tokyo, 1-1-1 Yayoi, Bunkyo-ku, Tokyo 113-8657, Japan

## Abstract

The distribution of inorganic elements in brown rice has been vigorously investigated for many years using the most advanced instruments of each era. The present study was a challenge to gain new insights into the distribution of various inorganic elements in brown rice by autoradiography using radioisotopes: ^22^Na, ^45^Ca, ^54^Mn, ^55^Fe, ^60^Co, ^63^Ni, ^65^ Zn, ^90^Sr, ^203^ Hg, and ^210^ Pb. Autoradiography of tissue sections using the Imaging Plate (IP) fully exploited its advantage of high-throughput imaging, enabling three-dimensional reconstruction that encompassed the entire brown rice grain. Consequently, characteristic distribution patterns of individual elements in the peripheral layer, endosperm, and embryo were identified following radiotracer supplementation to the culture solution. For instance, ^63^Ni was uniformly distributed within the endosperm during the early stages of development but progressively accumulated in the outer layers and embryo as growth advanced; such a pattern was not observed for ^54^Mn or ^55^Fe. To minimize the cost of the experiment, a direct injection method into the node was developed. This approach successfully visualized ^203^ Hg, demonstrating that its entry into the embryonic tissue is severely restricted irrespective of the developmental stage of the rice grain.

## Graphical Abstract

**Graphical Abstract:**
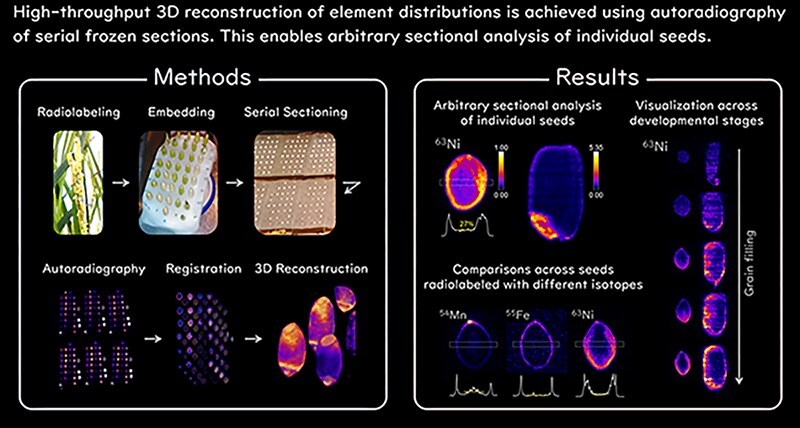

## Introduction

The accumulation of essential minerals and toxic metals in edible plants is closely related to human health. This is especially significant for food staples, including rice [1–4]. The total content of various elements in rice grain can be surveyed usually by the elemental analysis tools such as the inductively-coupled-plasma (ICP) spectrometry and atomic absorption spectrometry. These methods can also provide some information about the profile of elemental distribution in brown rice, for example, by comparing the elemental concentration before and after polishing. Several studies demonstrated that the bran layer and the embryo, which are easily milled off, had higher concentration of phosphorus (P), potassium (K), manganese (Mn), and iron (Fe) [5–7]. On the other hand, the concentration of cadmium (Cd) showed little change after polishing [8, 9], indicating an even distribution of this toxic metal throughout the grain, including the endosperm. The temporal changes in the accumulation of 14 elements in brown rice during grain-filling stages were also characterized, and the results suggested that there can be three patterns of elemental accumulation [10]. Then, to obtain more detailed information on the spatial distribution of each element, visualization techniques will be useful.

Elemental mapping using micro-focused X-ray fluorescence microscopy (XFM) is probably the analytical method that has provided the most data to date. Gu et al. conducted XFM and found that P, K, Mn, and Fe were indeed accumulated highly in the outer layer of the brown rice and embryo, while Cd distributed broadly in the endosperm. In more detail, P, K, and Fe distributed in the outer layer of the brown rice and embryo, while Mn accumulated preferentially in the embryo [11]. Ren et al. (2023) also performed XFM analysis and found that calcium (Ca), K, and Mn accumulated in the husk and pericarp/aleurone layer [10]. Elemental mapping with XFM further showed that sulfur (S), cupper (Cu), and zinc (Zn) distributed similarly in mature rice grain, being highest in the aleurone/pericarp and gradually decreasing from the outer parts of the endosperm toward the inner central part [10, 11]. In the embryo tissues, co-localization of Zn and Fe in the scutellum, and accumulation of Zn and Mn in the plumule were presented by XFM [12, 13]. Other elemental analyzers, including laser ablation ICP mass spectroscopy (LA-ICP-MS) and scanning electronic microscopy coupled with an energy dispersive X-ray spectroscopy (SEM-EDS), have also been applied to investigate the elemental distribution in rice grain [9, 14, 15]. The advantage of these analytical methods lies in their capability for multi-elemental mapping on the same cross-section; however, data collection from numerous samples remains challenging. In fact, most studies have reported elemental mapping data from a single cross-section derived from only a few rice grains.

Tracer experiments using radioisotope effectively evaluate elemental dynamics. By determining the radioactivity in the selected cells or organs, the transport characteristics of the labeled element can be quantitatively analyzed. The radiotracer experiment has a long history as an analytical method in various research fields including microbiology [16–19] and plant physiology [20–23]. By modifying the administration of radioactive tracers to plants, we can chase the elemental dynamics at specific time periods or among specific tissues while maintaining the plant in steady-state conditions [24–26]. The distribution map of the radio-labeled molecules can be acquired by the technique called autoradiography. If radioisotopes which emit positron or any radiation with high energy enough to transmit through plant tissue can be available, even real-time imaging in living plants can be achieved by sequential autoradiography with some specialized detectors [26–29]. Currently, however, the predominated autoradiography equipment is the commercially available Imaging Plate (IP). The IP has the advantage that the two-dimensional image data can be obtained at high throughput by arranging many samples within a size of, for example, 20×25 cm (BAS-IP MS 2025 E, GE Healthcare). Once elemental distribution data are obtained from multiple serial sections, these data can be reconstructed into three-dimensional images [30].

Therefore, autoradiography using radiotracers is expected to provide unique and valuable information on the accumulation of inorganic elements in brown rice, extending beyond what can be obtained from a single cross-section at maturity. In this study, radiotracer experiments were conducted to visualize the distribution of several essential elements, trace elements, and toxic metals in brown rice grown under normal hydroponic conditions, and to evaluate the effectiveness of autoradiography in plant metallomics research.

## Materials and methods

### Rice plant cultivation

Rice seeds (*Oryza sativa* L. var. Nipponbare) were surface sterilized and then germinated in tap water. The seedlings were cultivated in the plant growth chamber (L/D = 10 h/14 h, 30 °C) with the half-strength Kimura B nutrient solution [31] prepared by supplementing tap water with nutrients. The standard values established for the tap water in Tokyo are 200 mg/L for Na, 20 µg/L for Ni, 0.5 µg/L for Hg, and 10 µg/L for Pb. The concentration of Sr and Co in the tap water was measured with ICP-MS as described previously [32], and found to be 77 and 0.036 µg/L, respectively. The first ear appeared 49 days after germination.

### Administration of radionuclides from roots

At the boot stage, approximately 5 to 10 days before flowering, four plants were transferred to 1 L containers and one of the radionuclides listed in Table 1 was added to the nutrient solution to start labelling. Thereafter, any decrease in solution volume was replenished with non-radioactive hydroponic solution every 2-3 days. In total, about 1 L was replenished before harvest. Rice plants were cultivated until harvesting season and the grains at 28 DAF (days after flowering) were sampled and stored in a deep freezer. Especially for samples labeled with nuclides with daughter nuclides, long-term storage can reduce the influence of daughter nuclides on the results. In this case, ^90^Sr was stored for 2 weeks and ^210^ Pb for 7 months before proceeding to the visualization step. For the rice plants labelled with either ^54^Mn, ^55^Fe, or ^63^Ni, the grains were harvested also at 3, 5, 6, 7, 9, 12, and 15 DAF.

**Table 1:** Summary of radionuclides and experimental setup.

		^54^Mn	^55^Fe	^65^Zn	^60^Co	^63^Ni	^90^Sr	^203^Hg	^210^Pb	^45^Ca	^22^Na
Root absorption	kBq/plant	2100	1500	140	280	600	20	180	5.6		
Exposure (Fig. 1)	days	11	11	11	11	11	11	11	11		
Exposure (Fig. 2)	days	2	2			2					
Carrier	μM	1.9.E-02	1.2.E-02	1.5.E-02	1.2.E + 00	2.1.E-02	5.6.E-02	1.1.E-01	9.5.E-06		
Concentration ratio		2.8.E-03	2.7.E-04	9.9.E-02	1.9.E + 06	6.3.E + 01	6.3.E + 01	4.5.E + 04	2.0.E-01		
Direct injection	kBq/plant	1500				420	20	180		2500	150
Exposure (Fig. 3)	days	11				2	11			2	11
Exposure (Fig. 4)	days							33			
Carrier	μM	4.4.E + 01				5.0.E + 01	1.9.E + 02	3.8.E + 02		2.0.E + 02	1.3.E + 00
Concentration ratio		6.6.E + 00				1.5.E + 05	2.1.E + 05	1.5.E + 08		1.1.E + 00	1.5.E-01

### Addition of radionuclides directly into the first node via the cotton thread

In order to minimize radioisotope consumption and radioactive liquid waste, we devised an alternative radiotracer application method. The flag leaf sheath covering the first node was carefully pulled apart so as not to damage it, and the first node was exposed. Then a cotton thread was passed through the nodal vascular anastomoses, located at the bottom of the first node, using a needle. The tip of the cotton thread was dipped in the 300 µl of nutrient solution containing a single radionuclide (Table 1), so that the solution was gradually absorbed directly into the first node through the thread. After all labelled solution was absorbed, 200 µl of the nutrient solution without radionuclide was immediately added to the cotton thread to flush out any remaining labelled solution. This direct injection method was carried out at 3 DAF, and the grains were sampled at 28 DAF. For imaging mercury, ^203^ Hg was injected at either 3 DAF or 6 DAF, and the labelled grains were sampled after 8 h, 24 h, and at 28 DAF. The grains were stored in a deep freezer.

### Autoradiography and 3D image construction with fresh-frozen grain sections

The procedure of making the fresh-frozen 5 µm thick sections and autoradiography and 3D image construction was described previously [30]. Briefly, the frozen grains were sliced into 5 µm thick transverse sections at -25 °C, and one section in every 20 sections was preserved for the autoradiography. The section samples were contacted to the Imaging Plate (IP BAS-TR, GE Healthcare) under -80 °C for exposure for the periods listed in the Table 1, and the autoradiogram with the resolution of 10 µm/pixel was produced by the FLA-5000 image scanner (Fujifilm). The autoradiogram data were loaded in the ImageJ software using ISAC Manager plugin and stacked to construct the three-dimensional image with the support of the StackReq plugin [33].

### Evaluation of the incorporation of the radionuclide into the endosperm

One transverse section image at the middle of the grain was used to quantitatively evaluate the distribution of the radionuclide. The profile of the signal intensity in the line with 0.3 mm width was generated using the line profiling tool in ImageJ software. Then, the average of the signals in the central 60% area of the segment between the left and right peaks was taken as the signal of the endosperm center. This value was converted to a relative value, with the maximum defined as 100%.

### Simulation analysis of the radiation imparting the energy to the Imaging Plate

The energy imparted to the IP BAS-TR by the radiation emitted by each nuclide was analyzed by simulation with the PHITS (Particle and Heavy Ion Transport code System) version 3.28 [34].

In the model, geometry of the IP BAS-TR was set as a 1 cm diameter cylinder with the following five materials were layered: plant section (5 µm), PPS film (1.2 µm), sensitive layer (50 µm), support layer (250 µm), and magnetic layer (80 µm). The energy imparted to the sensitive layer was considered to contribute to create the radiation image. As the radiation source, the line segment of 5 µm length with 0 µm thickness was set and placed in the center of the plant section. Radiation was generated from a random position on this line segment in a random direction. Radiation type was classified into six groups: α-rays, β-rays, γ-rays, electron beams other than β-rays (conversion electron and Auger electron), photon rays other than γ-rays (characteristic X-rays), and positrons. For each group, 100 000 particles were generated, and the energy imparted to the photosensitive layer by the particles was calculated. The energy distribution of each group of radiation emitted from each nuclide was set up using the radioisotope source simulation function of PHITS. In the case of nuclides having daughter and granddaughter nuclides, the contribution of all nuclides present in equal amounts was calculated.

## Results

### Element-specific distribution to the periphery, inside the endosperm, and to the embryo was detected in the mature brown rice

**Figure 1:**
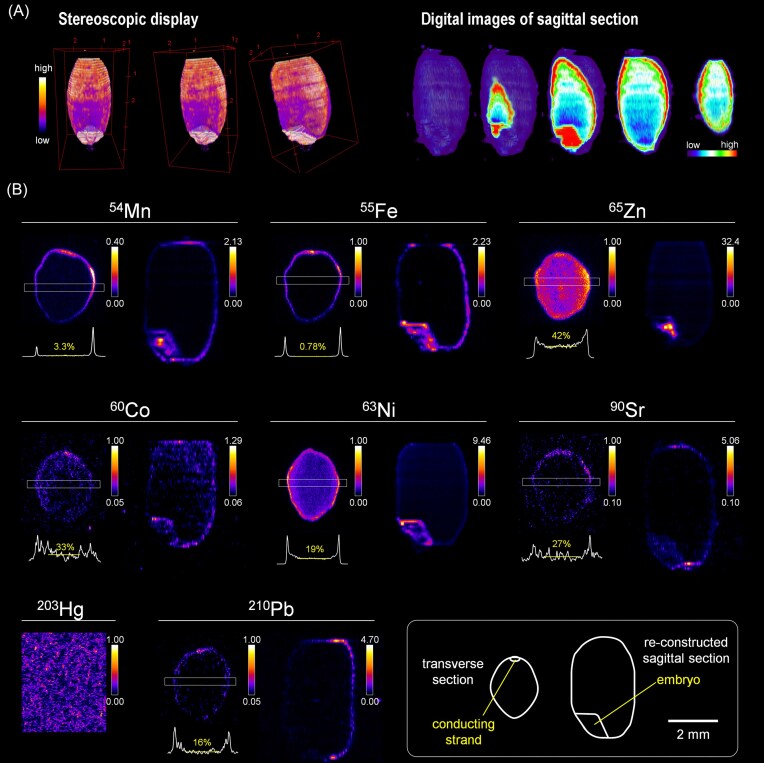
distribution of radionuclides absorbed from roots in mature brown rice. (A) example of three-dimensional reconstructed data using images of transverse sections of < sup > 63</sup > Ni. the 3D viewer displays the image three-dimensionally with transparency, so that the distribution of radionuclide both on the surface and inside the grain can be seen. the stereoreconstructed data can be used to digitally display the distribution of radionuclide in a sagittal section at any location.(B) the transverse section image (left) and the sagittal section image (right) made from the middle of the rice grain at 28 DAF.Note that the autoradiography of the same radionuclide will have different scales in the transverse- and sagittal-section images. the rectangle (0.3 mm width) superimposed on the transverse section image corresponds to the area used for generating the line profile presented below the image. the percentage presented in the line profile is the average value for the center of the endosperm (indicated by a yellow horizontal line), where the maximum value is set to 100%.

The distribution of ^54^Mn, ^55^Fe, ^65^ Zn, ^60^Co, ^63^Ni, ^90^Sr, ^203^ Hg, and ^210^ Pb absorbed from the roots after the booting stage in mature brown rice was investigated. Three-dimensional reconstruction of sequential autoradiographs allowed digital visualization of element distribution in sagittal sections (Fig. 1A). Section images can be obtained at any position (Fig. 1A), but in this paper, we will present one sagittal section including the embryo created at the center of the brown rice.

A sharp, strong signal of ^54^Mn was localized in the outermost layer of brown rice (Fig. 1B), which includes the cross cells, the tube cells, the seed coat, and aleurone layer [30]. Among the outer perimeter, there was a region of particularly high signal intensity on the upper side (i.e. the dorsal side). The ^54^Mn signal was also detected in the endosperm uniformly, but the average signal intensity was as low as 3.3% of that in the outermost layer (Fig. 1B). Like ^54^Mn, ^55^Fe was thinly localized in the outermost layer of brown rice, particularly in large amount on the upper part. However, ^55^Fe signal was almost absent in the endosperm, meaning that the average signal intensity at the center was less than one-hundredth of that at the outer edge of the brown rice (Fig. 1B). In contrast, relatively small differences in ^65^ Zn signal intensity across the sample indicated that Zn was distributed rather evenly throughout the entire brown rice (Fig. 1B). Looking at the signal profile, it was found that the signal intensity of ^65^ Zn gradually increased toward the outer part of the brown rice, yet the differences in signal intensity between the central part and the outer part was only about 2-fold (Fig. 1B). When comparing the distribution of the three radionuclides ^65^ Zn, ^54^Mn, and ^55^Fe in the embryo, ^65^ Zn and ^54^Mn exhibited a more pronounced concentration toward the embryo center than ^55^Fe (Fig. 1B), consistent with previous reports [12, 13].

For ^60^Co, the signal was barely detected. Although the signal was too weak to clarify, Co could be distributed throughout the brown rice and localized slightly richer toward the outer edge of the rice (Fig. 1B). General appearance of ^63^Ni distribution image was similar to that of ^65^ Zn, while the sharp peaks at the outer layer of the brown rice were analogous to those of ^54^Mn and ^55^Fe (Fig. 1B). A pattern of accumulation surrounding the embryo tissue was also noticed (Fig. 1B). The average intensity of ^63^Ni signal in the endosperm central region was 19% of the ^63^Ni intensity at the peaks (Fig. 1B). The signal of ^90^Sr was detectable only barely at the outer part of the brown rice, and the radiation of ^203^ Hg was not detected at all (Fig. 1B). One possible solution is to increase the amount of radiotracers administered. However, it should be noted that the ^203^ Hg radioisotope reagent contained significantly high concentration of stable Hg isotope, 2.35 mg/ml, as a carrier. One needs to be cautious about the idea of applying more ^203^ Hg, because increasing the amount of radiotracer can result in increased Hg concentration in the culture medium to toxic levels. Another matter unique to radioisotopes should be considered in the imaging of ^210^ Pb, which decays to ^210^-bismuth (Bi)(half-life: 5.01 days) and further to ^210^-polonium (Po)(half-life; 138 days). Therefore, although the autoradiographs of the grain doped with ^210^ Pb indicated that Pb tended to localize at the peripheral part of the brown rice (Fig. 1B), some influence of the radiation emitted by ^210^Bi and ^210^Po on the distribution images should be considered (see below).

### Radiation contributions and daughter nuclide issues identified by PHITS

Since the IP cannot discriminate between radionuclides, autoradiograms after ^210^ Pb administration may represent distributions of two daughter radionuclides, ^210^Bi and ^210^Po, in addition to ^210^ Pb. Therefore, aiming to consider which radiation contribution is significant in the visualization of the ^210^ Pb, as well as the nuclides used in Fig. 1B (Table 2), we performed PHITS simulation. PHITS is a general-purpose Monte Carlo particle transport simulation code capable of realistically simulating the behavior of radiation and particles. In this study, we used it to computationally estimate the amount of energy deposited in the sensitive layer of the IP by individual radiation particles emitted from the radioisotopes in the sample. The results showed that in the visualization of ^210^ Pb, α-rays emitted from ^210^Po accounted for 98.4% of the signal. Here, it is important to note that the image produced by radiation emitted from ^210^Po does not necessarily indicate “the dynamics of Po in plant”. In fact, there should be two types of ^210^Po in brown rice: those absorbed as ^210^Po and transported through the rice plant (presenting the dynamics of Po), and those that reach the brown rice as ^210^ Pb and decay to ^210^Po during frozen storage or exposure to the IP (presenting the dynamics of Pb). In our experiment, the ^210^ Pb solution had a radionuclidic purity of > 99% at the time of purchase and we added it to the roots one month later. Thus, the amount of ^210^Po at the time of addition was about 10% of ^210^ Pb. Then, in 7 months after harvest, this ^210^Po decayed to 35%. Therefore, we can estimate that the amount of ”^210^Po presenting the dynamics of Po” was significantly small comparing to the amount of ”^210^Po presenting the dynamics of Pb”. If the distribution of Po and Pb is to be explored, it can theoretically be achieved by administering a radioisotope in equilibrium with ^210^Po and ^210^ Pb, preparing sections right after sample collection, and then taking autoradiograms multiple times over several months.

**Table 2:** Simulated contributions of individual radiations to imaging

Nuclides	Half life	Radiations	Energy (MeV)	yield	contribution
^54^Mn	312 d	γ	8.35E-01	9.99E-01	32.9%
		X	5.42E-03	2.19E-01	66.9%
		ce	8.29E-01	2.23E-04	0.1%
^55^Fe	2.74 y	X	6.49E-03	2.72E-01	100.0%
^65^Zn	244 d	γ	1.12E + 00	5.00E-01	7.1%
		X	8.91E-03	3.82E-01	72.8%
		β+	1.42E-01	1.42E-02	20.1%
		ce	1.11E + 00	8.28E-05	0.0%
^60^Co	5.27 y	γ	1.33E + 00	9.99E-01	2.5%
		γ	1.17E + 00	9.98E-01	
		β-	9.58E-02	9.98E-01	97.5%
^63^Ni	100 y	β-	1.74E-02	1.00E + 00	100.0%
^90^Sr	28.8 y	β-	1.96E-01	1.00E + 00	49.9%
^90^Y^a^	64.1 h	β-	9.32E-01	9.99E-01	50.1%
		ce	1.74E + 00	1.02E-04	0.0%
^203^Hg	46.6 d	γ	2.79E-01	8.15E-01	1.7%
		β-	5.79E-02	1.00E + 00	65.3%
		ce	1.94E-01 ∼ 2.79E-01	1.83E-01	28.6%
		Auger	5.52E-02	4.76E-03	
		X	7.08E-02 ∼ 8.49E-02	1.21E-01	4.3%
		X	1.03E-02	5.38E-02	
^210^Pb	22.2 y	ce	3.02E-02 ∼ 4.65E-02	7.58E-01	0.3%
		β-	1.62E-02	1.60E-01	0.0%
		X	1.08E-02	2.26E-01	0.1%
		γ	4.65E-02	4.25E-02	0.0%
^210^Bi^a^	5.01 d	β-	3.89E-01	1.00E + 00	1.2%
^210^Po^b^	138 d	α	5.30E + 00	1.00E + 00	98.4%

a daughter nuclide.

b granddaughter nuclide

For ^90^Sr, its daughter nuclide ^90^Y contributes 50.1% (Table 2); however, since ^90^Y (half-life 64.1 h) is mostly generated after harvest, autoradiographs of ^90^Sr should essentially represent Sr dynamics in rice.

^60^Co, ^54^Mn, and ^65^ Zn emit high-energy photon rays, but it was beta rays and low-energy photon rays of a few keV that contributed to imaging (Table 2). This may explain why the IP BAS-TR produced high-contrasting distribution images in brown rice (Fig. 1B), whereas the radiography with nuclides that emit high-energy photon rays is sometimes thought to result in poor resolution.

### Nickel accumulation follows a distinct process compared to Fe and Mn

Having identified differences in the distribution of Mn, Fe, and Ni in mature brown rice, we then performed additional imaging experiment to determine the processes that cause these differences. Here, in addition to the mature brown rice, we visualized the distribution of ^54^Mn, ^55^Fe, and ^63^Ni in growing brown rice at 3 to 15 days after flowering (DAF), as well as 28 DAF.

**Figure 2:**
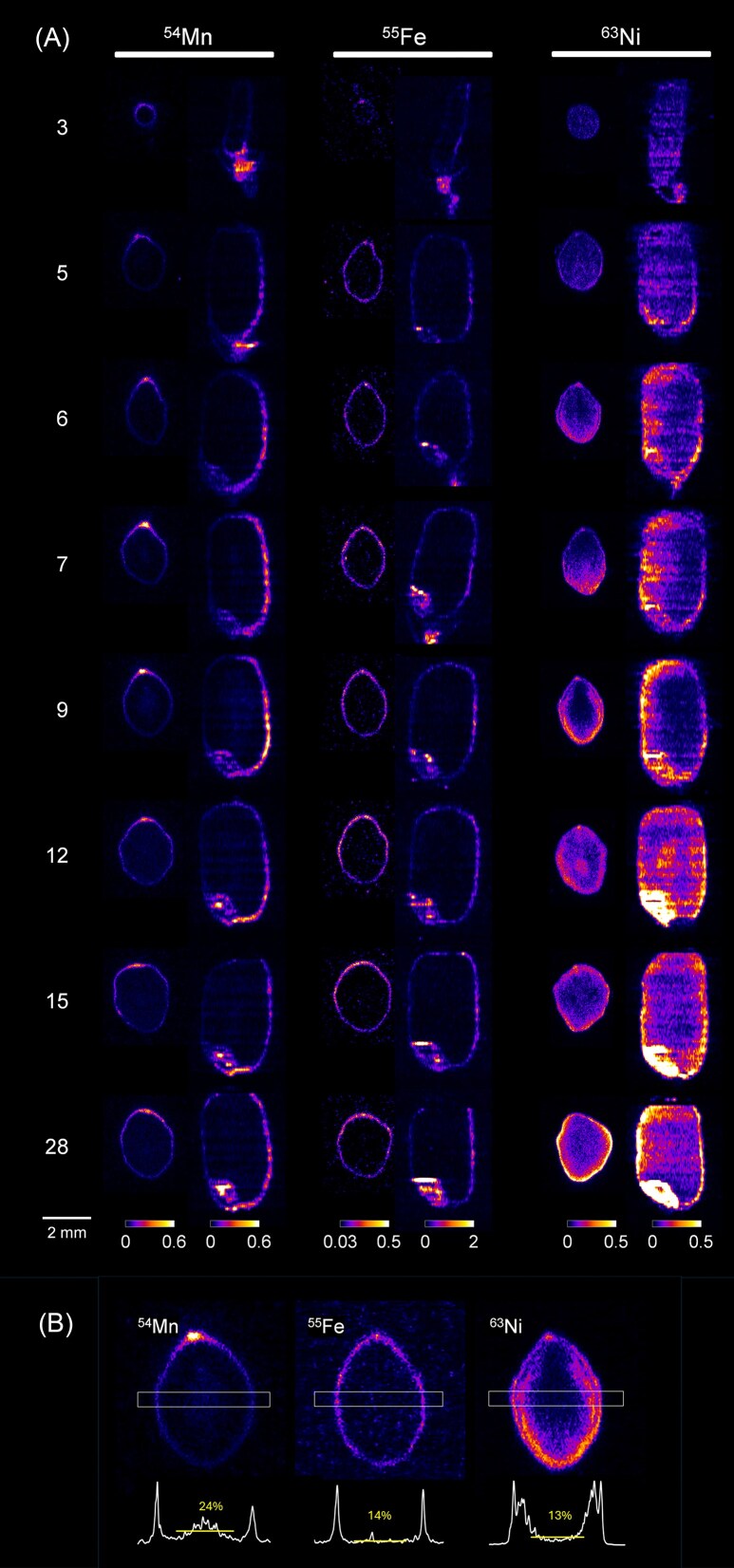
Changes in the distribution of Fe, Mn, and Ni over the course of rice growth from 3 DAF to 28 DAF (A) and the line profiles at 9 DAF (B). In (A), the transverse section image (left) and the sagittal section image (right) made from the middle of the rice grain were presented. In (B), the number in the center of the line profile is the average value for the center of the endosperm (indicated with yellow horizontal line) expressed as 100% of the higher peak on the brown rice surface.

It was found that both ^54^Mn and ^55^Fe were highly accumulated in the outermost layers of brown rice and in the embryo throughout the rice maturing process (Fig. 2A). In the outermost layers, both ^54^Mn and ^55^Fe were preferentially distributed in the upper half, i.e. on the dorsal side of the brown rice around the dorsal vascular tissue (Fig. 2A). In the endosperm, ^54^Mn signal appeared to be detected in the central region between 7 DAF and 12 DAF (Fig. 2B), and thereafter, ^54^Mn was distributed almost uniformly. Compared to ^54^Mn, the proportion of ^55^Fe distributed in the endosperm remained low over the entire development period (Fig. 2A). Unlike ^54^Mn and ^55^Fe, ^63^Ni was distributed throughout the brown rice until 5 DAF, with no localization to the surface area (Fig. 2A). Detailed observation revealed that in brown rice at 6, 7, and 9 DAF, ^63^Ni signal accumulated in the outermost layers and in an area slightly inside the aleurone layer (Fig. 2B). The two close peaks—the outermost layer and slightly inside the aleurone layer—became indistinguishable after 12 DAF, and at the same time intensive ^63^Ni accumulation to the embryo appeared to start (Fig. 2A). In the endosperm, ^63^Ni was unevenly distributed on the ventral side at 6 DAF and 7 DAF (Fig. 2A). Subsequently, accumulation of ^63^Ni in the center area of the endosperm was observed at 12 DAF, then its distribution changed to surround the outside of the endosperm without dorsal or ventral polarization (Fig. 2A).

### Devising a new method for adding radiotracers to the grain

**Figure 3:**
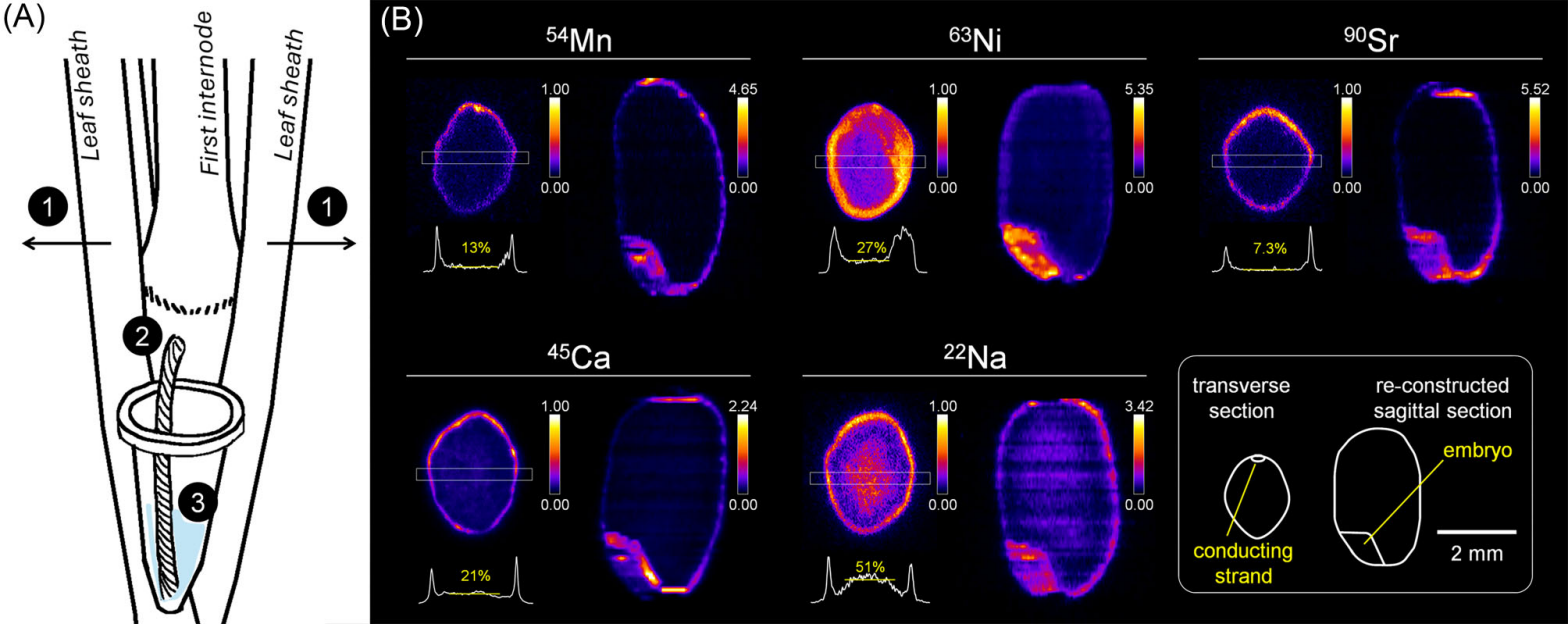
depiction of pulse addition of radionuclides via the thread through the first node (referred to as the direct injection method) at 3 DAF (A), and the distribution of the injected radionuclides in the mature grain (B). in (A), ① the leaf sheath was pulled apart to expose the first node, and ② the cotton thread was penetrated at the bottom part of the first node. ③ the plastic tube with the injection solution was attached with medical tape, and the tip of the cotton thread was dipped in the solution. in (B), the transverse section image (left) and the sagittal section image (right) made from the middle of the rice grain were presented for each radionuclide. the rectangle (0.3 mm width) superimposed on the transverse section image indicates the area used for generating the line profile presented below the image. the percentage value presented in the line profile is the average signal intensity for the center of the endosperm (indicated with yellow horizontal line) compared to that at the higher peak on the brown rice surface.

It was quite imaginable that not a small amount of the radiotracers added to the hydroponic solution would remain there or distribute to the roots, leaves, stems, and thus did not work as the radiation source for the autoradiography in the grain. To overcome this issue, we designed a more economical and simpler method for tracer loading in which radioactive solution was injected directly into the nodal vascular bundles (Fig. 3A). In this study, the 300 µl solution labeled with the radionuclide and the subsequent 200 µl non-labeled solution were all absorbed from the node within 2 hours.

The ^54^Mn signal was higher around the periphery of the brown rice at 28 DAF, especially around the dorsal vascular tissue, and the average signal intensity at the central part of the endosperm was 13% of that at the periphery peak (Fig. 3B). The ^63^Ni signal decreased gradually from the outside toward the center of the endosperm, reaching 27% (Fig. 3B). Clear images of ^90^Sr distribution in the brown rice were obtained, whose appearances were quite similar to the ^54^Mn images. The ^90^Sr signal intensity at the outer part of the brown rice was more than 10-fold of that in the endosperm. Overall, the distribution characteristics of ^54^Mn, ^63^Ni, and ^90^Sr absorbed from the node (Fig. 3B) were consistent with those absorbed from the roots (Fig. 1B), though quantitative differences in the endosperm were noted (see Discussion).

With this injection method, the distribution of Ca, the chemical analogue of Sr, was also visualized for comparison. It was found that ^45^Ca was highly concentrated in the periphery of the brown rice and was also diffused uniformly throughout the endosperm (Fig. 3B). This feature was common to ^90^Sr. But the relative signal intensity in the endosperm was 21% for ^45^Ca, which was relatively higher than that found in the autoradiography of ^90^Sr (Fig. 3B). Additionally, this method was sufficient to visualize even sodium (Na), a light element that is more difficult to detect with SEM-EDX and XFM. The distribution pattern of ^22^Na was similar to many other elements in that it was highly accumulated in the periphery of the brown rice and in the embryo, but uniquely exhibited higher signal at the center of the endosperm than in the outer parts (Fig. 3B).

### Mercury distribution has been successfully visualized by the new method

**Figure 4:**
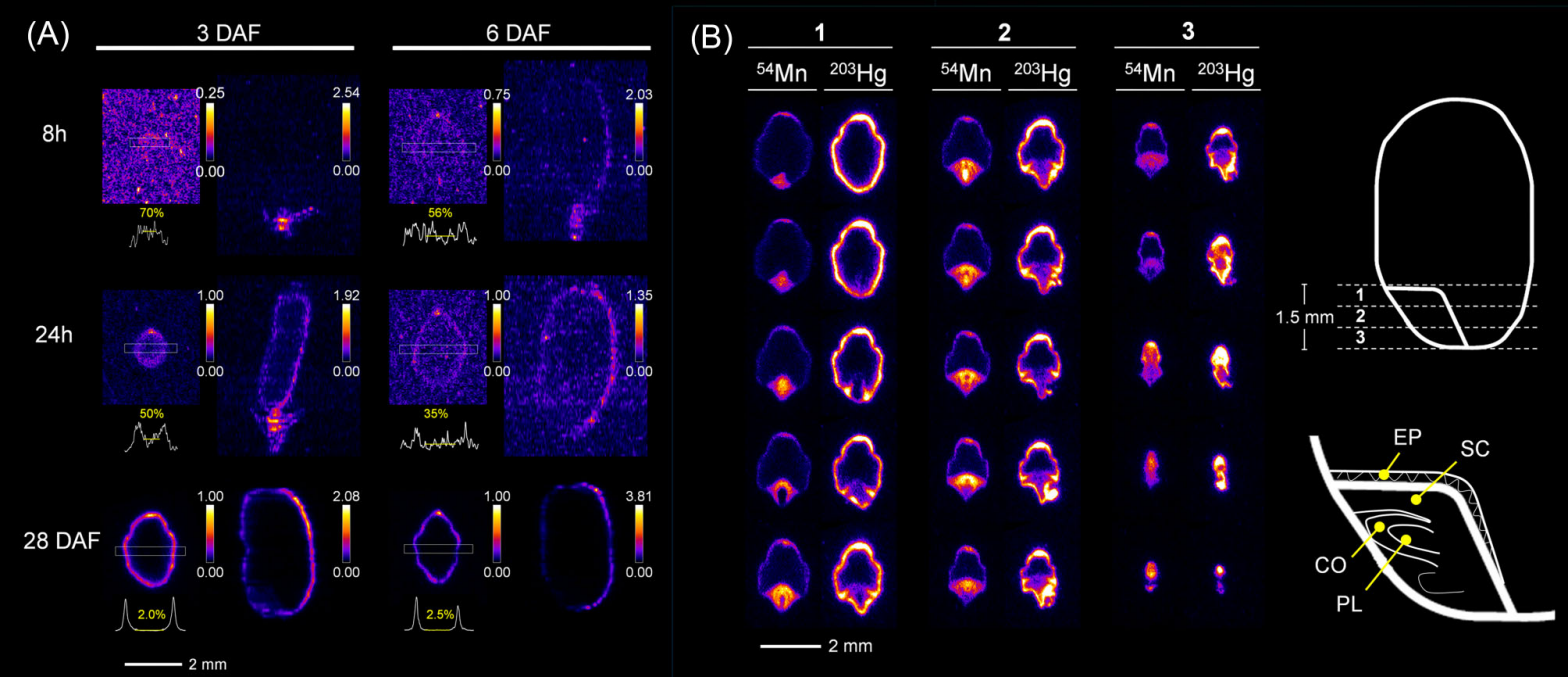
Autoradiographs of < sup > 203</sup > Hg in the growing rice grain at maturity (28 DAF), and 8 hours and 24 hours after injection from the node at 3 DAF and 6 DAF (A), and the comparison of < sup > 54</sup > Mn and < sup > 203</sup > Hg accumulation around the embryo at 28 DAF (B). The rectangle (0.3 mm width) superimposed on the transverse section image indicates the area used for generating the line profile presented below the image. The percentage value presented in the line profile is the average signal intensity for the center of the endosperm (indicated with yellow horizontal line) compared to the higher signal in the surface region. In (B), the 15 transverse section images covering the entire embryo tissues were presented. The sequential data of < sup > 54</sup > Mn is extracted from the same data presented in Fig. 3B. The position of the sections (region 1, 2, 3) was described in the illustration on the right. The structure of the embryo was also presented. EP: epithelium layer, SC: scutellum, CO: coleoptile, PL: plumule.

Challenges were made to visualize ^203^ Hg, which was not distributed to the brown rice in the amount needed for visualization when it was applied to the roots. After injecting ^203^ Hg to the node at 3 DAF or 6 DAF, the distribution of ^203^ Hg in the growing caryopsis was examined 8 hours later and 1 day later, and also at maturation stage. As early as 8 hours after injection, ^203^ Hg reached the brown rice and was distributed on the dorsal side of the outer layer. At maturity, ^203^ Hg was localized around the outside of the brown rice and was absent in the endosperm. The characteristic feature of ^203^ Hg distribution in terms of exclusion from the embryo was more easily understood when compared to other elements, e.g. ^54^Mn (Fig. 4B). The accumulation of ^54^Mn in the embryo was remarkable. In the embryo, the signal of ^54^Mn was especially high in the regions that are thought to be the scutellum and the plumule (Fig. 4B). On the contrary, ^203^ Hg was deposited at the boundary between the embryo and endosperm, possibly at the bottom ends of the epithelium layer (Fig. 4B).

## Discussions

In this study, by using commercially available and widely used autoradiography tool: Imaging Plate, we attempted to discover the characteristic distribution of inorganic metals in brown rice that have attracted attention in the current plant research field. Once the radiotracer-dosed samples were taken, the pre-exposure process simply involved making sections and contacting them to the IP under low temperature. The image scanning process took less than an hour for one imaging plate, no matter how many sections were on it. This high-throughput imaging is one of the advantages over other mapping methods. For brown rice at maturity, about 50 autoradiography per grain can be acquired at once to obtain a three-dimensional view of the elemental distribution providing a great deal of information. The autoradiography has the resolution of 10 µm per pixel at highest, which is significantly poor compared to other techniques that allow multi-elemental bioimaging at the cellular level [35]. Still, autoradiography using IP described here can be a practical method for capturing the overview of the elemental distribution at the tissue level in plants. We expect that 3D visualization will yield valuable insights into organs characterized by complex structures, such as nodes in stems and rhizoids in roots.

In this study, radionuclides added to the roots just before flowering were found to reach the growing caryopsis by 3 DAF at the latest and produce radiographs thereafter (Figs 1 and 2). And these images of each metal in mature rice grain conserved the characteristics of the elemental distribution maps in the paddy samples, podding mix-culture samples, and sand-culture samples visualized by X-ray fluorescence [10, 13, 36, 37], e.g. the accumulation of Mn and Fe particularly in the bran layer, and the relatively high Zn concentration in the endosperm. Additionally, the high concentration of Mn in ovular vascular trace has been shown by the quantitative X-ray fluorescence [38]. Our data support this and further reveal that such Mn distribution in outermost layers is maintained throughout the maturation process of brown rice (Fig. 2). The characteristic of Fe, whose overall distribution pattern did not change throughout the maturation process, was distinct from Mn (Fig. 2) and also from Zn [30], which temporarily accumulated in the center of the endosperm during the maturation process. The storage of Fe and Zn in various tissues in rice grains is thought to depend on their binding property to nicotianamine (NA), 2′-deoxymugineic acid, and phytic acid, but much remains to be elucidated [36, 39]. Ion transporters also seem to contribute to the establishment of element-specific distribution patterns. For Zn, the tonoplast localizing transporter OsMTP1 highly expresses in aleurone and embryo to specifically accumulates Zn in these tissues [40]. The transportation of Fe from endosperm to embryo tissue can be mediated by the group I Yellow Stripe 1-Like (YSL) family transporters [41–44]. Among these OsYSLs, OsYSL2 showed transport activity also for Mn(II)-NA, and the participation of this transporter in Mn dynamics has been suggested [41, 43].

Nickel is the most common identifiable cause of metal-related contact dermatitis [45] and prevalence of Ni allergy is known to be 14.5% in the general population in EU [46]. Since the Ni intake could cause a flare-up of allergic Ni dermatitis in dose-dependent manner [47], low-Ni diet will benefit the Ni sensitive patients [48].

In mature brown rice, Ni is known to be highly concentrated in bran [6, 49]. However, the accumulation of Ni, like Zn, in the surface layer of brown rice relative to the endosperm is not as pronounced as for Fe and Mn [50]. In the embryo, elemental mapping by LA-ICP-MS showed that Ni accumulates in the periphery, while Zn accumulates in the center [51]. These characteristics of Ni distribution in mature brown rice were reproduced in our data (Fig. 1B). In addition to these, our experiments have shown that Ni distribution is highly variable during the growth process of brown rice. Particularly interesting is the fact that the distribution of Ni in 6 DAF and 7 DAF, which are in the milk stage, is very similar to the distribution of Cd in the same period [30]. The similarity in the distribution of Cd and Ni was also previously reported based on the synchrotron-based XFM analysis in mature brown rice [52]. However, by examining element distribution across the whole grain, including the embryo, during maturation, we identified a clear difference: beyond 12 DAF, Ni showed substantial incorporation into the embryo (Fig. 2A), while Cd exhibited limited entry [30]. It is possible that both elements initially move through brown rice by a common transport system, then there may be a mechanism that sorts out Ni and Cd in the process of migration from the endosperm to the embryo. In addition, in the endosperm of mature brown rice, Cd accumulates in a ring-like pattern surrounding the central region [11, 30], whereas Ni does not (Fig. 2A), suggesting that their dynamics within the endosperm are not identical.

In plant research, the molecules involved in uptake and root-to-shoot transport of Ni have been gradually identified [53–55]. However, the membrane Ni transport system functioning in seed tissues is largely unknown. For Fe, NA is significantly involved in the phloem transport and loading in seeds [56]. Similar to Fe, Ni is thought to form stable complexes with NA and migrate long distances as Ni(II)-NA [57–59], but our radiotracer study revealed that its behavior at the brown rice, the terminus of phloem transport, is quite different from that of Fe and other metals (Fig. 2). Given that various nickel-containing enzymes have been found in plants [60], the distribution of those enzymes in grains may be one of the determinants of Ni accumulation patterns.

If radiotracer dosing around flowering time can be used to monitor the pattern of elemental dynamics in brown rice, it would be less costly in terms of money and labor than continuously dosing throughout the growing season. Radioactive ions injected directly into the node for a short period of time probably accumulated rapidly in the tissues around the node and were then gradually introduced into long-distance transport over a long period of time, resulting in the similar distribution pattern within the mature seed as the radioisotopes that were absorbed by the roots. However, when the accumulations of ^54^Mn, ^63^Ni, and ^90^Sr in the endosperm were quantitatively evaluated, there were slight differences in the values obtained by each of the two methods (Fig. 1B, Fig. 3B). The relative signal intensity of ^54^Mn at the endosperm, for example, was 3.3% when ^54^Mn was supplied from the root (Fig. 1B), while it was 13% in the direct injection method (Fig. 3B). The cause of this difference is not determined at this time, but one possibility is that the amount of radiotracer introduced into the long-distance transport system rapidly decreases in the direct addition method. If this were to occur in reality, the distribution of radioisotopes flowed into the brown rice at earlier stages would be overemphasized in the radioactivity images acquired at maturity. Returning to the issue of ^54^Mn, its value of 24% at 9 DAF (Fig. 2) and 3.3% at 28 DAF (Fig. 1B) indicates that the degree of distribution to the endosperm is higher for the earlier growth stage. Therefore, the higher value of 13% found by the direct introduction method (Fig. 3B) agrees with our hypothesis. The dynamics of ^63^Ni should be opposite to ^54^Mn. In summary, the distribution pattern of metal elements in the brown rice can be determined with the transient direct introduction of radiotracers around the time of flowering, although the result will not be in perfect agreement with the one obtained with radiotracers absorbed from the roots over a longer period of time. The supply of radiotracer from the root would also progressively decline over time with this approach, and the rate of decline would likely differ among elements. To sustain a constant supply, continuous flushing of the hydroponic solution containing radionuclide would be ideal; however, this must be balanced against the increased cost of radioactive waste.

Additionally, the direct injection method could circumvent certain physiological regulatory mechanisms in plants. For instance, it is presumably not suitable for reproducing the distribution of elements such as carbon, phosphorus, and sulphur, that are metabolized and change their chemical forms in the plant cells. It is entirely plausible that the chemical form converted differs depending on whether these elements enter shoot cells or enter root cells via the normal absorption pathway. Chelation is another form of metal transport within the vascular bundle. The major ligands for Mn and Ni in the phloem can be NA, as well as carboxylic acids [61, 62]. In this study, autoradiograph images showed no particular difference in the distribution characteristics of ^54^Mn and ^63^Ni added from the roots and those directly introduced into the node vasculatures. Additionally, the results that ^63^Ni and ^54^Mn accumulated on the outside of the brown rice, and the extent of accumulation was more pronounced for ^54^Mn, are consistent with the quantitative ICP measurement data reported previously [63]. Therefore, it is possible that ^54^Mn and ^63^Ni applied from the node moved through the vascular bundles in the same form as those transported from the roots, but this cannot be determined at this time.

It is known that Hg can make complex with thiol-containing biomolecules such as phytochelatins and glutathione. In xylem sap, Hg was detected as a form of Hg-phytochelatins [64]. Here, direct injection method succeeded in capturing a pattern of ^203^ Hg accumulation that closely resembles the distribution of S, which accumulates on the periphery of brown rice and is difficult to access inside the endosperm and embryo tissues [11]. The closely analogous distribution of ^203^ Hg and S indicates that the major form of Hg in brown rice may be in complex with sulfur-rich molecules. Indeed, previous studies have suggested that inorganic mercury accumulates in bran and may exist as cysteine complexes, whereas approximately 80% of methylmercury is distributed in white rice [65]. Therefore, we consider that the ^203^ Hg captured in this experiment likely represents the dynamics of inorganic mercury.

One key advantage of high-sensitivity tracer experiments using radioisotopes is the feasibility of pulse-chase experiments, in which a labeled substance is absorbed for a brief period to track its subsequent dynamics. Although the two application experiments in this study were not designed in this manner, the signal intensities derived from the obtained images should allow for accurate estimation of the required application time for each element. On the other hand, there are limitations in the radiotracer study, primarily the restriction to single-nuclide analysis. Additionally, unlike XFM, the lack of simultaneous surface visualization makes it challenging to precisely overlay autoradiographs with histological images at the cellular level.

Finally, autoradiography also allowed us to show the ^22^Na distribution in brown rice. The Na concentration in brown rice is very low, only about 1% or less of that in leaves [66]. In such tissues, it is not easy to analyze the elemental distribution using X-ray fluorescence, because the detection sensitivity of light elements including Na is not high. Since autoradiography has a completely different detection principle, it could be used complementary to other detection methods when one aims to collect information on various elemental distributions in plants.

## Conclusion

Autoradiographic imaging using the Imaging Plate successfully reproduced the element-specific distribution patterns in brown rice that have been reported by other elemental mapping methods. Furthermore, due to the high throughput of autoradiography, three-dimensional analysis of elemental distributions and their temporal changes—such as those of ^54^Mn, ^55^Fe, and ^63^Ni in this study—can be performed efficiently. In this context, radiation-based imaging represents an effective and versatile approach that can contribute substantially to metallomics research, serving as both a complementary and compatible technique alongside other analytical methods. When conducting an experiment, it is essential to carefully consider the effects of issues specific to radiotracer experiments, such as daughter nuclides, carriers, and the method and dose of radiotracer applied, on the results. The experimental conditions and discussions presented here will serve as a useful reference for researchers who wish to try radiotracer experiments in the future.

## Data Availability

All data of this study are available in the main text.
